# The impact of artificial intelligence with multi-head self-attention based deep learning model for students' academic performance monitoring

**DOI:** 10.3389/frai.2026.1904923

**Published:** 2026-09-03

**Authors:** Mahmoud Ragab

**Affiliations:** 1 Information Technology Department, Faculty of Computing and Information Technology, King Abdulaziz University, Jeddah, Saudi Arabia; 2 Mathematics Department, Faculty of Science, Al-Azhar University, Cairo, Egypt

**Keywords:** artificial intelligence, deep learning, metaheuristic optimization algorithms, multi-head self-attention, students' academic development

## Abstract

**Introduction:**

Educational data mining has been applied to examine students' data collected from different educational organizations to forecast academic students' performance, which could assist them in accomplishing improved results in their upcoming courses.

**Methods:**

This study presents the impact of artificial intelligence with multi-head self-attention on students' academic development using metaheuristic optimization algorithms (IAIMSA-SADMOA) model. The study aims to advance an effective method for students' academic performance using artificial intelligence tools to improve learning outcomes, engagement, and personalized education. The min-max normalization is employed in the data normalization phase for transforming input data into a beneficial format. The fruit fly optimization algorithm is deployed for the feature selection process to select the most related features from a dataset. Moreover, the proposed model designs bidirectional long short-term memory and multi-head self-attention mechanisms for the student's academic performance classification process. The parameter tuning process is performed through a sparrow search algorithm to enhance the classification performance.

**Results and Discussion:**

The experimental evaluation of the IAIMSA-SADMOA technique occurs using a benchmark dataset. The empirical results indicated the enhanced performance of the proposed method in comparison with recent approaches.

## 1 Introduction

The steady rise in education data is because of the constant production of data from diverse sources, namely learning management systems, e-learning, student feedback analysis systems, and admission systems (Robert et al., 2024). The student data attained from the above-mentioned source is utilized to make a simple query-level decision, while large data remains unutilized because of the intricacy and data variability. In the learning process, student performance is a significant part. Predicting student output is crucial to identify learners who may show lower academic results in the future (Yousafzai et al., 2021). This information is useful and is used in prediction if that data is processed into knowledge. Consequently, this knowledge might improve the excellence of teaching and assist students in achieving their academic aims. Educational data mining (EDM) employs data mining methods to analyze data produced from educational backgrounds (Hussain et al., 2021). Additionally, using EDM aids policymakers in planning how to advance student performance. Therefore, it will boost the learning and experience of the students in the academy.

The student performance prediction is a vital task that is examined through EDM (Nabil et al., 2021). This task forecasts the value, which describes the student's results, marks, grades, and so on. Predicting student success, failures, and attrition rates are the key topics addressed in the literature review of this research study (Yagci, 2022). Every person in this field seeks an early alert system to anticipate learning in the initial phases. This early warning system decreased the education expenses and demands on time and location. Presently, development in technology, especially the artificial intelligence (AI) domain, have considerably impacted numerous educational aspects (Lau et al., 2019). One of the most prominent revolutions is seen in teaching and learning practices, in which AI-driven learning techniques have arisen as an encouraging way to improve education methods. Such state-of-the-art methodologies utilize the strength of AI models to customize learning experiences, adjust to specific student requirements, and offer practical feedback, thus possibly transforming conventional educational models (Parkavi et al., 2024).

AI tools are transforming conventional teaching approaches, altering the learning experience, and offering fresh paths for customized learning. With progressions in machine learning (ML) paradigms, data analytics, natural language processing (NLP), and AI, it can increase student engagement, enhance learning performances, and deliver personalized assistance to distinct students (Raja et al., 2024). Incorporating AI in student life comprises numerous applications, automatic grading systems, virtual assistants, etc. This AI-based tool can adjust to learners' specific learning styles, abilities, and preferences and offer tailored support, content recommendations, and feedback. The AI's impact on students' learning goes beyond the classroom (Pertiwi et al., 2024). AI-based academic support devices provide continuous support over chatbots and virtual advisors, assisting in course selection, responding to FAQs (frequently asked questions), and providing academic counseling. The rise of ML, especially deep learning (DL) methodologies, has significantly improved prediction performance in numerous fields, including business, health, agriculture, etc. This encourages the implementation of DL in educational data (Taher, 2023).

### 1.1 Role of generative artificial intelligence in educational ecosystems

The prompt adoption of generative artificial intelligence (GenAI) tools, like large language models, transforms educational practices by intelligent tutoring, content generation, and supports personalized learning. Hence, future educational prediction methods should consider the feasible impact of GenAI-based learning on student behavior and academic achievements. Recent studies on AI-adoption and the abilities of large language models furthermore emphasize the GenAI in decision-support and educational systems (Ji et al., 2024). AI, particularly GenAI, has transformed education using intelligent content generation, personalized learning, and AI-based learning settings. Current investigations emphasize the increasing role of ChatGPT and LLMs in improving student engagement, academic performance monitoring, and learning outcomes, whereas also increasing challenges related to assessment practices and academic integrity (Mittal et al., 2024).

The prompt advancements of GenAI, particularly in large language models (LLMs), has considerably transformed the educational ecosystems by supporting content generation, automated feedback, intelligent tutoring, and personalized learning. They also initiate new challenges in data mining, as AI-assisted learnings may influence traditional academic performance indicators like GPA and grades. The proposed framework supports this objective by presenting an intelligent prediction model, while future studies may use GenAI-based learning behaviors to further improvements in model adaptability and prediction accuracy.

### 1.2 Paper contribution

This study proposes an impact of artificial intelligence with multi-head self-attention on students' academic development using metaheuristic optimization algorithms (IAIMSA-SADMOA) model. At the initial stage, the min–max normalization is deployed in the data normalization step. Next, the fruit fly optimizer algorithm (FOA) is used for the selection of the feature procedure. Besides, the proposed model designs bidirectional long short-term memory and multi-head self-attention (BiLSTM-MSA) mechanisms for the student's academic performance classification process. Eventually, the parameter tuning process is performed through a sparrow search algorithm (SSA). The experimental evaluation of the given model occurs using a benchmark dataset. The novelty of the proposed architecture lies in the combined use of SSA for the hyperparameter optimization and FOA for feature selection along with a BiLSTM-MSA classifier. Contrary to existing methods that optimizes only single stage, the proposed architecture jointly optimizes model parameters and feature selection, which results in high prediction performance in student academic performance classification.

## 2 Literature survey on students' academic development

In Nayani et al. (2025), a new meta-heuristic hybrid galactic rider swarm optimizer (GRSO) system is designed. A hybrid DL is applied for the prediction process, in which the classifier outcome has been enhanced using the GRSO system. Now, the convolutional neural network (CNN) and Recurrent Neural Networks (RNN) hyperparameters are enhanced through the GRSO system. The pre-processing stage is implemented with outlier removal and the data-filling technique. Balachandar and Venkatesh (2025) proposed a novel method called the multi-dimensional student performance prediction (MSPP), which is motivated by sophisticated data pre-processing and feature selection models utilizing DL. Feng et al. (2025) developed a student's academic performance prediction model depending on students' classroom behavior images (SCBI). This approach intends to combine SCBI, essential data, and academic detail to offer detailed student academic performance prediction. This research comprises training three DL algorithms for extracting student behavioral features as negative and positive.

Silpa et al. (2024) addressed the concerns by leveraging ensemble ML models to predict success rates of students over the “Academic Success Predictor” (EML-ASP) model. This model gathers and pre-processes data from the UCI repository, using Boosted, Bagged, RUS Boosted, and Improved Ensemble paradigms for training and testing. This model displays higher performance with a subset of key features. Malik and Jothimani (2024) presented FeatureX, a hybrid methodology for selecting efficient factors that influence student performance and decrease school dropout levels. FeatureX incorporated filter- and wrapper-based approaches to capture appropriate features to predict student performance.

Babu et al. (2023) presented an innovative metaheuristic optimizer-based feature sub-set selection with DL (MOFSS-ODL) approach for student performance prediction. Furthermore, the presented approach utilizes an outlier detection method. Additionally, the CBOA is employed to select relevant features with lower complication and higher performance. Following that, a sailfish optimization with stacked sparse autoencoder (SFO-SSAE) methodology is leveraged for educational data classification. Apriyadi and Rini (2023) aimed to design a predictive method of student performance with SVRA. The hyperparameter optimizer technique utilized is the metaheuristic algorithm (MA). Once the SVR hyperparameter is achieved, then, a student performance predictions model with dual modules PSVR modeling and GSVR modeling is achieved. Nadar (2023) suggested a modified XGBoost (MXGB) approach to predict student performance by stream-based examination. An adapted form of the XGBoost model employs cross-validation to boost its performance. Shaikh and Güngör (2025) presented a study representing that there was a sharp rise in AI-related educational research, particularly in 2024. In the Scopus database, the term AI emerged as the most prevalent keyword, appearing 44 times. It was followed by higher education and ChatGPT.

## 3 Algorithm and system design

**Figure 1:**
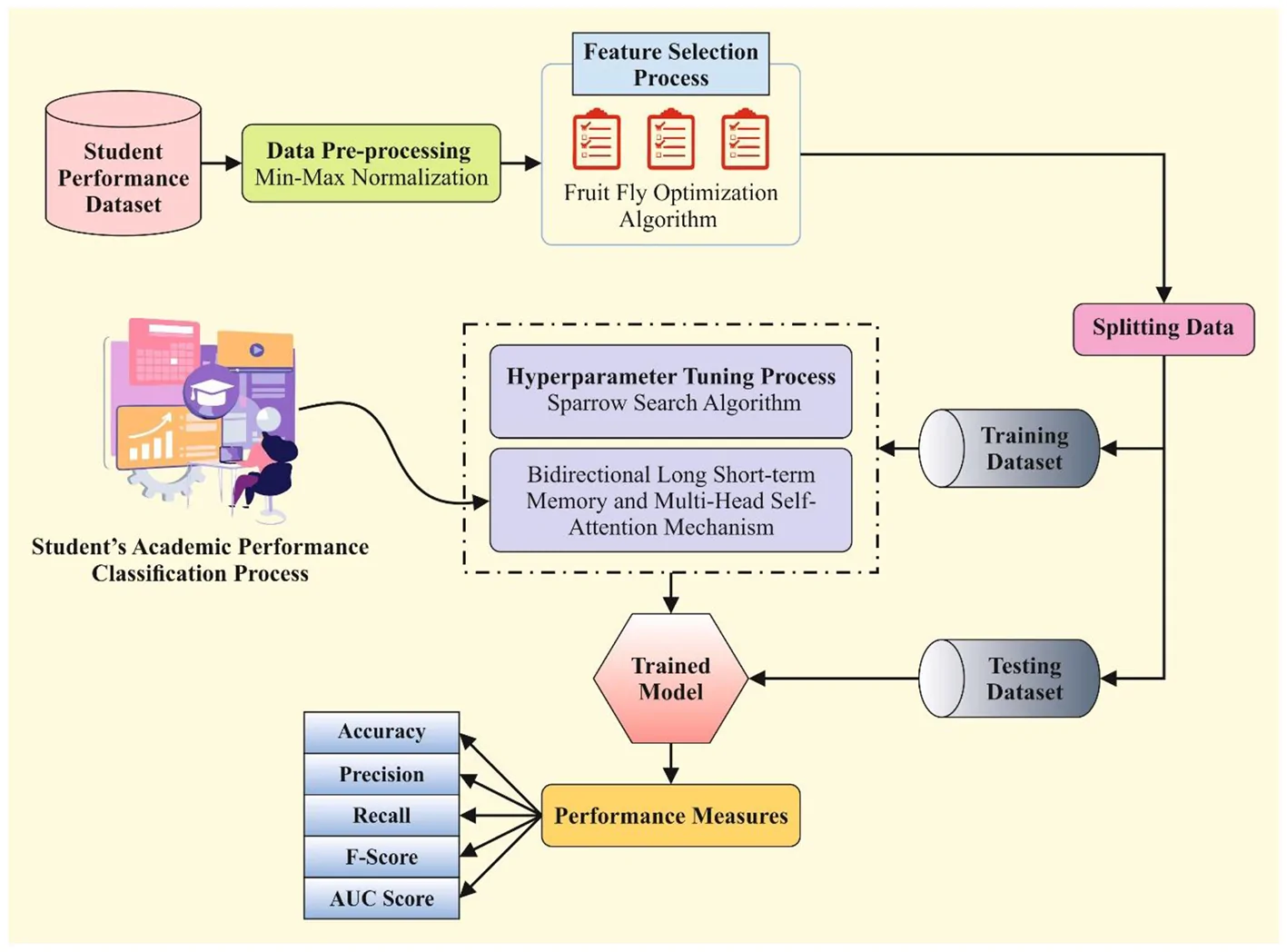
Complete procedure of IAIMSA-SADMOA model.

This study proposed an IAIMSA-SADMOA model. It aims to advance an effective method for students' academic performance using AI tools to improve learning outcomes, engagement, and personalized education. It comprises data pre-processing, feature selection, classification, and hyperparameter tuning. Figure 1 depicts the complete process of the proposed model.

The proposed IAIMSA-SADMOA model is performed sequentially. Initially missing values are handled, followed by the encoding of categorical features. The dataset is divided into 80% of training, 20% of testing and 70% of training and 30% of testing. Min–max normalization is performed to the training data, and the same transformation is utilized for the testing data, FOA-based feature selection to recognize important features. The SSA algorithm tuning the BiLSTM-MSA hyperparameters, and the optimized model is trained and evaluated through the testing data. *K*-fold cross validation is further performed to evaluate the reliability of the proposed method.

### 3.1 Data pre-processing through normalization

Primarily, the min–max normalization is employed for transforming input data into a beneficial format. It carries out linear transformation of original data consequently creating a balance of value contrasts (Shaikh and Güngör, 2025). This methodology might employ the Equation 1:


$$\begin{array}{l} X_{new}=\frac{X-\min\left(X\right)}{\max\left(X\right)-\min\left(X\right)} \end{array}$$ (1)

*X*_*new*_ refers to novel value from the normalized outcomes; *X* signifies old value; *Max*(*X*) and *Min*(*X*) indicate maximal and minimal values in the database.

### 3.2 . Feature reduction using FOA model

Following by, the FOA is deployed for selecting features to choose the most related features from a dataset. The FOA is an inclusive optimizer model inspired by the fruit fly's searching behavior (Henderi et al., 2021). The FOA was selected because of its computational simplicity, effective global search capability and fast convergence. In contrast with generally used optimization methods, it provides an effective balance between exploitation and exploration by reducing the redundant features and preserving the discriminative information.

Fruit flies have a distinctive pattern in recognizing spoiled fruit and they fly toward the food based on vision and smell. An optimizer model imitates social behavior and search patterns of fruit flies. The FOA discovers the ability of a global optimizer as the best solution. This model is a simpler approach for easily upgrading its features and leads to easy implementation and provides an outstanding optimum solution. Therefore, FOA is used to resolve several real-time problems. The fruit fly's searching behavior discovers possible solutions for numerous optimizer difficulties. The fly's population is generated at random, and a fitness function (FF) has been applied to assess and evaluate the quality according to the category of the optimizer issue as provided in Equations 2 and 3.


$$\begin{array}{l} X_{i}=X_{\alpha xis}+R_{v} \end{array}$$ (2)

$$\begin{array}{l} Y_{i}=Y_{\alpha xis}+R_{v} \end{array}$$ (3)

Whereas, the *i*^*th*^ searching agent was signified as *X*_*i*_, *Y*_*i*_ represents the top location for each of the flies. *R*_*v*_ refers to a random position or value applied by all flies. The distance calculation between the food position and the source is shown in Equations 4 and 5.


$$\begin{array}{l} Distance_{i}=\sqrt{X_{i}^{2}+Y_{i}^{2}} \end{array}$$ (4)

$$\begin{array}{l} S_{i}=\frac{1}{Distance_{i}} \end{array}$$ (5)

The concentration judgment value calculation *S*_*i*_ at every location of fruit flies and the smell concentration rate *Smell*_*i*_ are specified using Equation 6:


$$\begin{array}{l} Smell_{i}=func\left(S_{i}\right) \end{array}$$ (6)

The *Best*_*smell*_ from the instance is characterized using Equation 7:


$$\begin{array}{l} \left[Best_{smell},\;Best_{index}\right]=\max\left(Smell\right) \end{array}$$ (7)

Upgrade the maximal concentration value of *x and y* coordinates, and upgrade the position of the best fruit using Equation 8.


$$\begin{array}{l} X_{\alpha xis}=X\left(Best_{index}\right) \end{array}$$ (8)

$$\begin{array}{l} Y_{\alpha xis}=Y\left(Best_{index}\right) \end{array}$$ (9)

FF deliberates the classifier accuracy and the chosen feature count. It increases the classifier accuracy and decreases the set dimension of the preferred feature. As a result, the succeeding FF is applied to estimate individual solutions as given in Equation 10.


$$\begin{array}{l} Fitness=\alpha *ErrorRate+\left(1-\alpha \right)*\frac{\#SF}{\#All\text{\_}F} \end{array}$$ (10)

**Figure 2:**
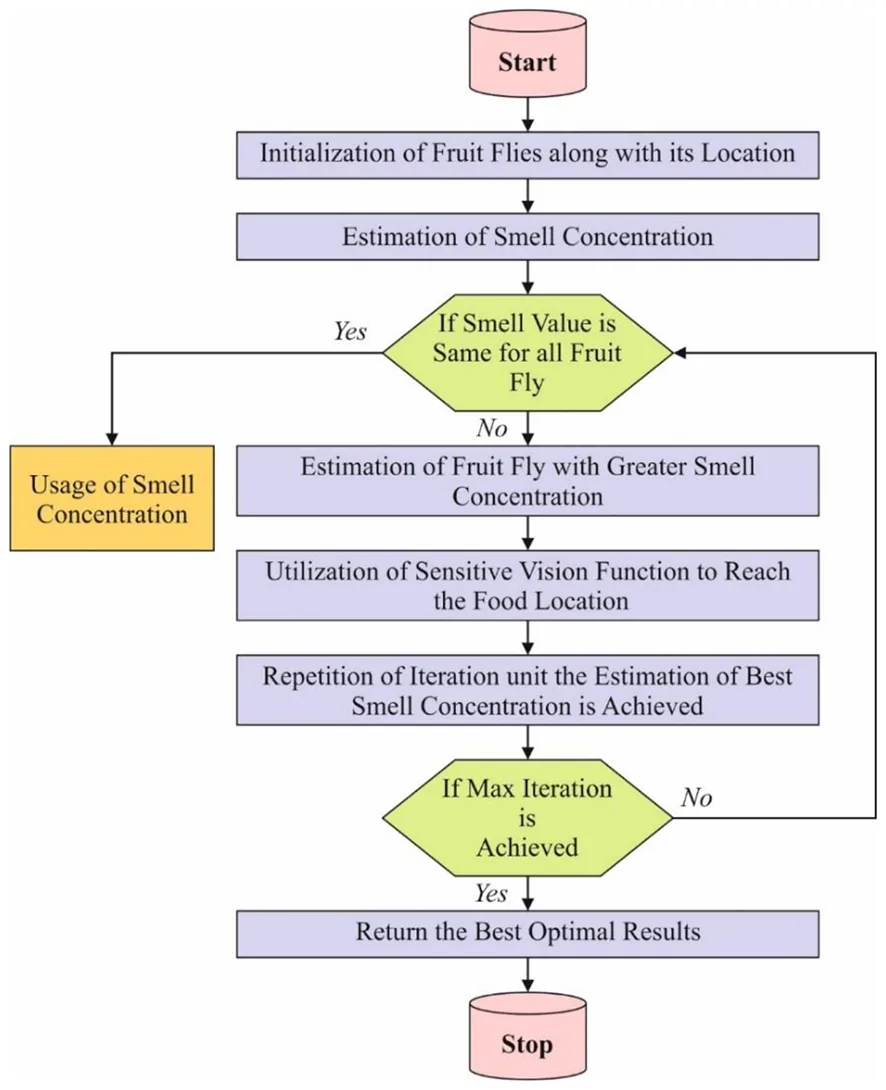
Flowchart of FOA.

Whereas, *ErrorRate* indicates the classification error rate employing the designated aspects. *ErrorRate* is calculated as the incorrect percentage among 0 and 1. *#SF* signifies selected feature counts and *#All*<*uscore*>*F* denotes complete feature counts in the novel database. α is applied to control the impact of classification quality and sub-set length. Figure 2 indicates the flowchart of the FOA.

### 3.3 BiLSTM-MSA for student's academic performance classification

Moreover, the presented model designs a BiLSTM-MSA mechanism for the student's academic performance classification process. LSTM neural networks are broadly employed to process time-series prediction concerns (Gangadevi et al., 2025). Bi-LSTM is an improved variant of LSTM that acquires more comprehensive data because of its ability to process time-series information in either forward or backward directions. Though the input data are tabular, the attributes are organized in the consistent order depending on their educational relevance, allowing the BiLSTM to capture contextual relationships between student features. Moreover, the MSA mechanism adaptively highlights the most important features, decreasing the influence of minimum significant features and enhancing the illustration for academic performance prediction. Every LSTM cell comprises various gates such as an input *i*, a forget *f*, an output *o*, and a memory cell *c*. The overall process of LSTM is given as: The sigmoid function σ() has been employed to control the data that will be discarded from the cell state over the forget gate and that data will be retained in the cell state over the input gate. The forget gate and input gate can be defined using Equation 11.


$$\{\begin{array}{c} f_{t}=\sigma \left(W_{f}X_{t}+R_{f}h_{t-1}+b_{f}\right)\\ i_{t}=\sigma \left(W_{i}X_{t}+R_{i}h_{t-1}+b_{i}\right) \end{array}$$ (11)

Subsequently, an innovative candidate will be attained from *h*_*t*−1_ and *X*_*t*_ through the tanh() layer using Equation 12.


$$\begin{array}{l} c_{t}=Mh\left(W_{c}X_{t}+R_{c}h_{t-1}+b_{c}\right) \end{array}$$ (12)

Afterward, the upgrade of the novel cell state will be carried out using Equation 13:


$$\begin{array}{l} c_{t}=f_{t}⊙C_{t-1}+i_{t}⊙c_{t} \end{array}$$ (13)

At last, the output data will be calculated by Equation 14:


$$\{\begin{array}{c} o_{t}=\sigma \left(W_{o}X_{t}+Rh_{t-1}+b_{o}\right)\\ h_{t}=o_{t}⊙tanh\;\left(c_{t}^{o}\right) \end{array}$$ (14)

Now *R*_*f*_, *Rj, R*_*c*_, and *R*_*o*_ indicate the recurrent weights of LSTM, *b*_*f*_, *bi, b*_*c*_, and *b*_*o*_ depict the biases of forgetting, input, memory cell, and output gates, *W*_*f*_, *Wj, W*_*c*_, and *W*_*o*_ refer to the weighted matrices of forgetting, input, memory cell, and output gates, correspondingly. ⊙ means Hadamard product. According to the above equation, the output of *t* th LSTM cell in the reverse LSTM chain is attained and specified *h*′(*t*). The hidden layer (HL) of Bi-LSTM equivalent to *t* th cell might be specified using Equation 15.


$$\begin{array}{l} H_{t}=\left[h_{t},{\;h}_{t}^{\prime }\right] \end{array}$$ (15)

**Figure 3:**
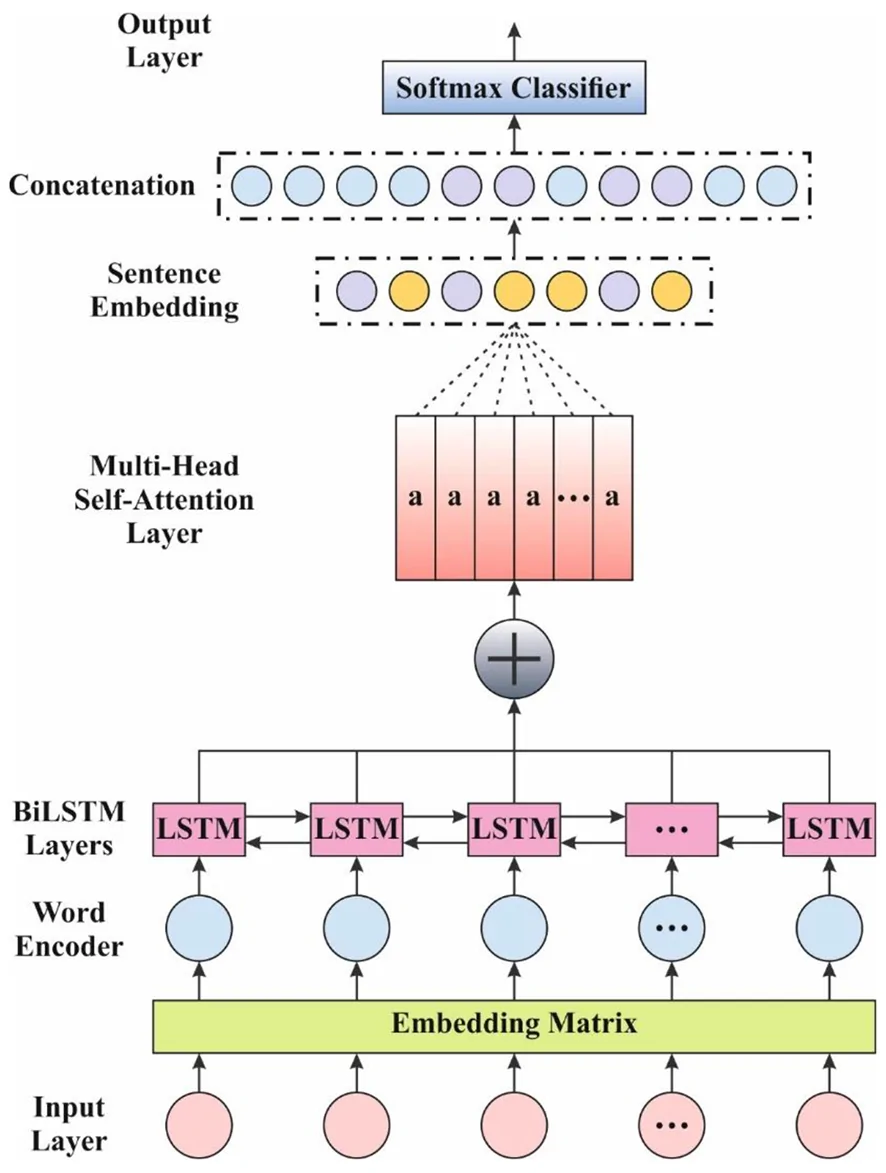
Structure of BiLSTM-MSA technique.

Now *W*_*h*_ indicates a weighted vector. Figure 3 depicts the structure of the BiLSTM-MSA technique.

### 3.4 Parameter tuning model

Eventually, the parameter tuning process is carried out through SSA to enhance the classification performance. The hyperparameters of an algorithm play an important role in its performance under improper circumstances for hyperparameters like the dropout rate, learning rate, recurrent unit counts, and convolutional kernel counts can result in underfitting or model overfitting (Li et al., 2025). The SSA was used for hyperparameter optimization because of its strong adaptive search mechanism and global optimization capability. It effectively balances the exploitation and exploration, enabling the BiLSTM-MSA model in identifying optimal hyperparameter configurations, also reducing the risks of premature convergence which may occur with conventional optimization methods.

Capture the learning rate as an instance: if set too high, the training process of the model may fail to converge; if set too low, the training speed turns out to be very slow. As a result, finding the best integration of hyperparameters guarantees the model is implemented well on either the testing or training sets.

Utilizing its low-complexity characteristics, swarm intelligence mechanism, and dynamic role transformation, SSA determines stronger adaptability and optimizer efficacy in time-series prediction. Its global searching ability successfully processes data non-stationarity, however, the dynamic adjustment mechanism guarantees the parameters of methodology associated with real-time distributions of data, offering precise and stable prediction assistance for real-world applications. After being combined with DL methods, SSA also improves its robustness and generalization, making it a significant device in the time-series analysis field. During SSA, explorers with improved fitness values select food achievement during the searching procedure. As an explorer has the responsibility to find food for the whole sparrow population and offers foraging ways for every follower, they get a larger foraging search area than followers. The location upgrade of explorers in all iterations is defined by Equation 16:


$$X_{i,j}^{t+1}=\{\begin{array}{c} X_{i,j}^{t}•\exp\left(-\frac{i}{\alpha •iter_{\max}}\right)\;R_{2}<ST\\ X_{i,j}^{t}+Q•L\;otherwise \end{array}\;$$ (16)

Now, *t* characterizes the present iteration number, *j* = 12, 3, ⋯*d*, and *Iter*_max_ refers to constant signifying the maximal iteration counts. *X*_*ij*_ specifies the location information of the *ith* sparrow in the *jth* size. α∈(0, 1) refers to randomly generated numbers. *R*_2_(*R*_2_∈[0, 1]) and *ST*(*ST*∈[0.5, 1]) characterize the warning value and security threshold, correspondingly. *Q* stands for an arbitrary number that follows a standard distribution, and *L* symbolizes *a*1*xd* matrix with each component set to 1. If *R*_2_<*ST*, it indicates that there are no hunters in the foraging habitat, and explorers can execute broad searching processes. On the other hand, if *R*_2_≥*ST*, it specifies that very few sparrows have identified predators and delivered alarms to the population, warning all sparrows to rapidly travel to safer places for foraging.

In the foraging procedure, some followers continually observe explorers. If they sense that explorers have discovered improved food sources, they instantly leave their present places to fight for food. The location update of followers can be explained by Equation 17:


$$X_{i,j}^{t,+1}=\{\begin{array}{c} Q\cdot \;exp\;\left(\frac{X_{worst}-X_{i,j}^{t}}{i^{2}}\right)\;i>\frac{n}{2}\\ X_{p}^{t+1}+|X_{i,j}^{t}-X_{p}^{t+1}|\cdot A^{+}\cdot L\;otherwise \end{array}$$ (17)

Now, *X*_*p*_ symbolizes the best location presently engaged by explorers, and *X*_*worst*_ epitomizes the present global poor place. *A* means 1 × *d* matrix where every element is arbitrarily allocated as 1 or −1, and *A*^+^ = *A*^*T*^(*AA*^*T*^)−1. If *i*>*n*/2, it specifies that the *ith* follower with a lower value of fitness has not gained food and is in an extreme hunger condition, therefore, has to fly to some other place for hunting. If danger is noticed, sparrows on the edge of the group rapidly travel to safer places to protected improved locations, however, sparrows in the middle of the group shift at random to halt closer to others. The location update of sparrows in this setting is designated using Equation 18:


$$X_{i,j}^{t+1}=\{\begin{array}{c} X_{best}^{t}+\beta \cdot \left|X_{i,j}^{t}-X_{best}^{t}\right|\;f_{i}>f_{g}\\ X_{i,j}^{t}+K•\frac{\left|X_{i,j}^{t}-X_{worst}^{t}\right|}{f_{i}-f_{w}+\varepsilon }\;otherwise \end{array}\;$$ (18)

Whereas, *X*_*best*_ embodies the present global best location. The step-size controller parameter β denotes randomly generated number following the standard distribution with a mean of 0 and a variance of 1. *K*∈ [−1, 1] refers to a randomly generated number, *f*_*i*_ symbolizes the fitness value, *f*_*g*_, and *f*_*w*_ represents present global best and poor fitness values, correspondingly, and ε means smaller constant to stop division by 0.

Here, the minimization of the classifier rate of error is measured as the FF, as set in Equation 19.


$$\begin{array}{lll} fitness\left(x_{i}\right) & = & ClassifierErrorRate\left(x_{i}\right)\\ \; & = & \frac{no\;of\;misclassified\;instances}{Total\;no\;of\;instances}*100 \end{array}$$ (19)

## 4 Results

The experimentation analysis of the IAIMSA-SADMOA system is tested under the student performance database (Dataset 1) (Dong et al., 2025) and student performance dataset (Dataset 2)^1^ Student performance database contains 2,392 samples under 0–4 grades. The dataset simulates the academic performance of students using demographic, behavioral, and educational factors comprising duration of study, attendance, parental involvement, and extracurricular participation. The dataset mainly involves secondary or high school students, making the student level equivalent to *K*−12/secondary education. The complete details of this database are shown in Table 1. The complete number of features is 14 but only 10 are chosen. Next, Predict Students' Dropout and Academic Success Dataset incorporate data from various institutional databases with demographic features, socioeconomic indicators, enrolment information, and academic performance records of undergraduate students enrolled in different programs including agronomy, design, education, nursing, journalism, management, social services, and technology-related disciplines. To provide a more extensive evaluation under class imbalance, *k*-fold cross-validation was utilized, and additional evaluation metrics, including Matthews correlation coefficient (MCC) and Kappa were used. These metrics provided more reliable evaluation of the classifier's performance than the overall accuracy alone while dealing with imbalanced datasets. Table 2 shows the detailed parameter settings of the proposed system. The experimentation was executed using Python 3.11 (Python Software Foundation, Wilmington, DE, USA) with a workstation equipped with an NVIDIA GPU, 16 GB RAM, and Windows 11 operating system. For experimental validation, 80:20 and 70:30 of training/testing dataset is employed. To avoid data leakage, all preprocessing operations that capture from the data, containing normalization, feature selection, and hyperparameter optimization, were performed utilizing only the training dataset. The captured normalization parameters and the selected feature subset was subsequently useful to the testing data without change. The final method performance was then validated on the unseen testing data utilizing accuracy, precision, recall, *F*1-score, MCC, and Kappa.

**Table 1:** Details of Dataset 1.

Grade	Description	No. of samples
0	“‘A' (GPA ≥ 3.5)”	107
1	“‘B' (3.0 ≤ GPA < 3.5)”	269
2	“‘C' (2.5 ≤ GPA < 3.0)”	391
3	“‘D' (2.0 ≤ GPA < 2.5)”	414
4	“‘F' (GPA < 2.0)”	1,211
Total samples		2,392

**Table 2:** Detailed parameter settings.

Component	Parameter	Value
Data preprocessing	Normalization	Min–max normalization
Feature selection	Optimization algorithm	FOA
	Population size	30
	Maximum iterations	100
	Fitness function	Classification error + selected feature ratio
	Selected features	10 (from 14 features)
Classification	Model	BiLSTM + MSA
	BiLSTM hidden units	128
	Number of BiLSTM layers	2
	Dropout rate	0.5
	Attention heads	8
	Activation function	ReLU
	Output layer	Softmax
Training	Optimizer	Adam
	Learning rate	0.001
	Batch size	32
	Epochs	100
	Loss function	Cross-entropy loss
Hyperparameter optimization	Algorithm	SSA
	Population size	30
	Maximum iterations	100
	Discoverer ratio	0.2
	Safety threshold (ST)	0.8
Dataset	Total samples	2,392
	Selected features	10
	Number of classes	5
Evaluation	Training/Test split	80:20 and 70:30
Performance metrics	Metrics	Accuracy, precision, recall, F1-score, AUC, MCC, kappa score

### 4.1 Student performance dataset (Dataset 1)

**Figure 4:**
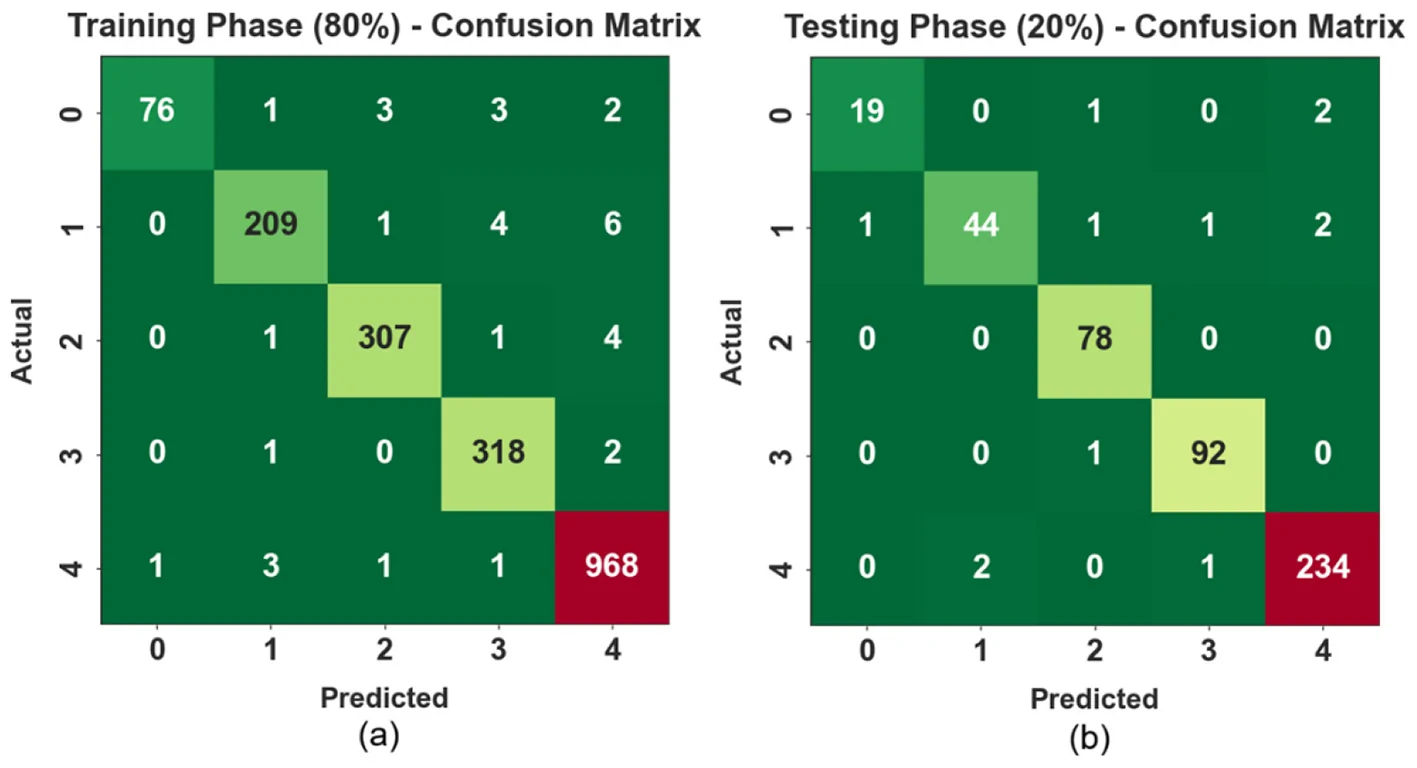
Confusion matrices **(a, b)** 80:20 of TRPHE/ TSPHE with Dataset 1.

Figure 4 demonstrates the confusion matrices for the 80% of training and 20% of testing set of the proposed method. In two sets, most instances are accurately classified, as specified by the maximum values beside the diagonal, whereas only a few samples are misclassified into different class labels. The training confusion matrix illustrates efficient learning of the training data, and the testing confusion matrices confirms that the method preserves high classification accuracy and better generalization on unseen data.

Table 3 depicts the students' performance prediction of the proposed approach under 80:20 of Training Phase (TRPHE)/Testing Phase (TSPHE). The result specified that the method has properly identified all the different class labels. Under 80% TRPHE, the proposed method obtains average *accu*_*y*_ of 99.27%, *prec*_*n*_ of 98.03%, *reca*_*l*_ of 96.19%, *F*_*Score*_ of 97.06%, and *AUC*_*score*_ of 97.82%. Moreover, on 20% TSPHE, the proposed method obtains average *accu*_*y*_ of 99.00%, *prec*_*n*_ of 96.63%, *reca*_*l*_ of 94.76%, *F*_*Score*_ of 95.63%, and *AUC*_*score*_ of 97.02%.

**Table 3:** Students performance prediction of IAIMSA-SADMOA model under 80%:20% of TRPHE/TSPHE on the Dataset 1.

Class labels	Accuracy	Precision	Recall	*F*-score	AUC score	MCC	Kappa score
TRPHE (80)							
0	99.48	98.70	89.41	93.83	94.68	93.68	97.26
1	99.11	97.21	95.00	96.09	97.32	95.60	
2	99.42	98.40	98.08	98.24	98.89	97.90	
3	99.37	97.25	99.07	98.15	99.25	97.78	
4	98.95	98.57	99.38	98.98	98.95	97.91	
Average	99.27	98.03	96.19	97.06	97.82	96.57	
TSPHE (20)							
0	99.16	95.00	86.36	90.48	93.07	90.15	96.30
1	98.54	95.65	89.80	92.63	94.67	91.88	
2	99.37	96.30	100.00	98.11	99.63	97.76	
3	99.37	97.87	98.92	98.40	99.20	98.01	
4	98.54	98.32	98.73	98.53	98.54	97.08	
Average	99.00	96.63	94.76	95.63	97.02	94.98	

**Figure 5:**
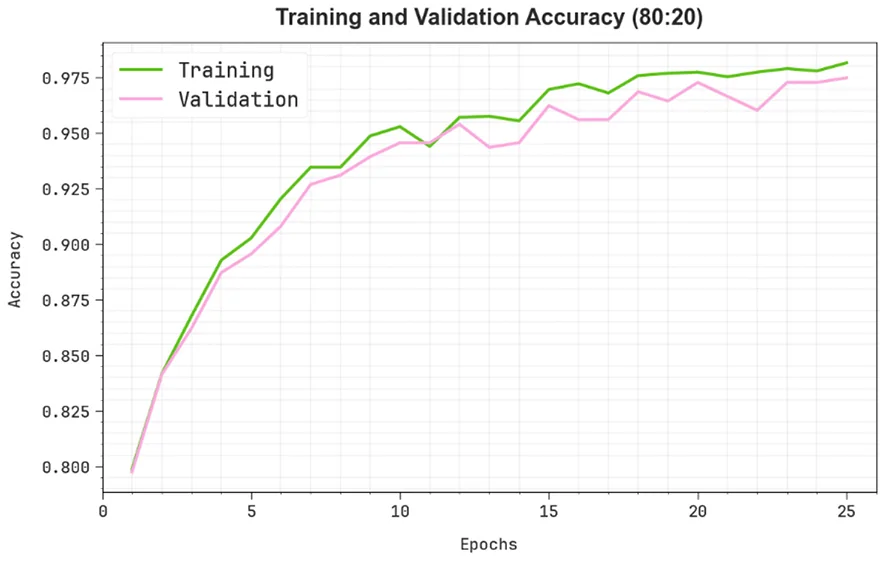
*Accu*_*y*_ curve of IAIMSA-SADMOA model under 80:20 with Dataset 1.

In Figure 5, the training (TRAN) *accu*_*y*_ and validation (VALD) *accu*_*y*_ performances of the proposed model under 80:20 is shown. The figure highlighted that the TRAN and VALD *accu*_*y*_ values demonstrate an upward tendency, indicating the efficiency of the proposed methodology with greater solution through numerous iterations. Moreover, the TRAN and VALD *accu*_*y*_remain close throughout the epochs, demonstrating lesser overfitting and revealing higher efficiency of the proposed approach.

**Figure 6:**
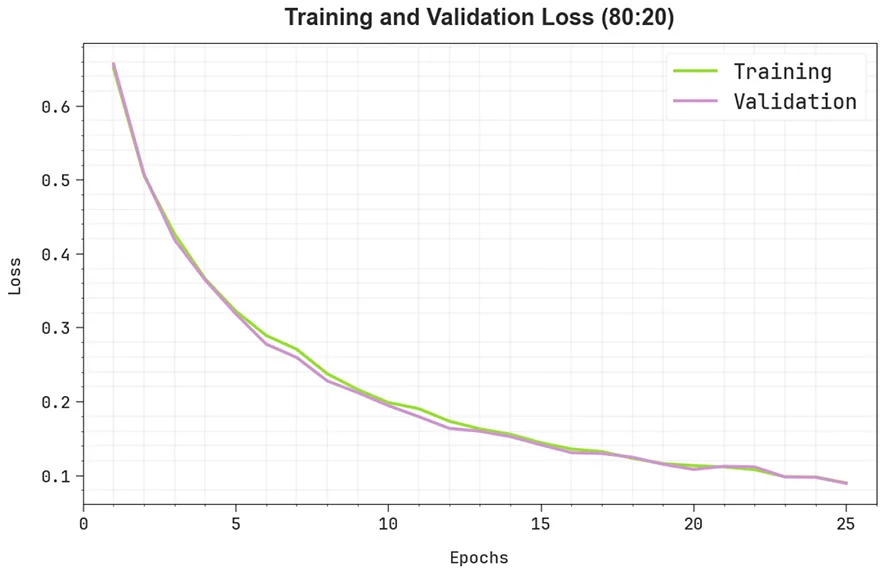
Loss curve of IAIMSA-SADMOA model under 80:20 on Dataset 1.

In Figure 6, the TRAN and VALD loss graphs of the proposed framework under 80:20 is depicted. It is noted that the TRAN and VALD values exemplify downward trends, reporting the effectiveness of the proposed approach in stabilizing a trade-off.

**Figure 7:**
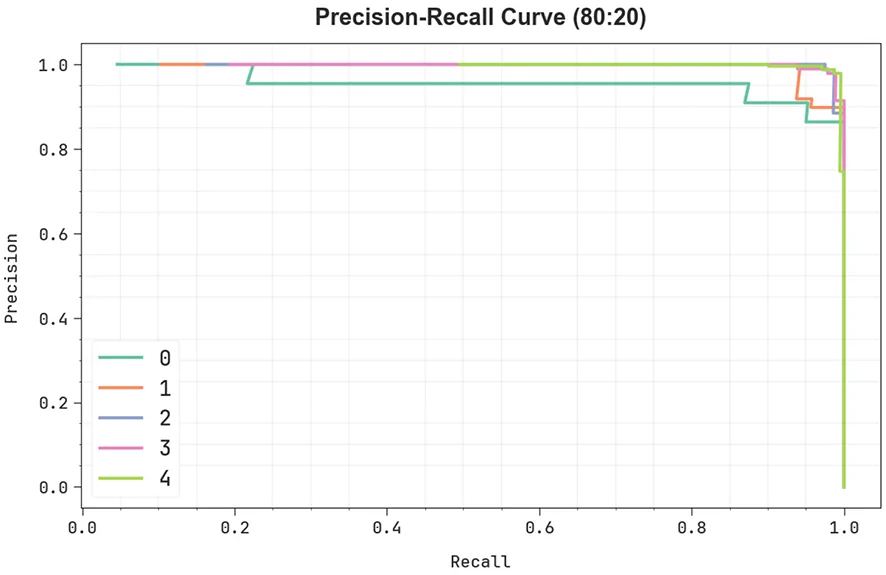
PR curve of IAIMSA-SADMOA system under 80:20 with Dataset 1.

In Figure 7, the precision-recall (PR) curve inspection of the proposed framework under 80%:20% provides clarification into its performance by mapping Precision against Recall for each class. The figure exposes that the proposed framework persistently achieves elevated PR values over assorted classes, specifying its proficiency to retain a major portion of true positive predictions.

**Figure 8:**
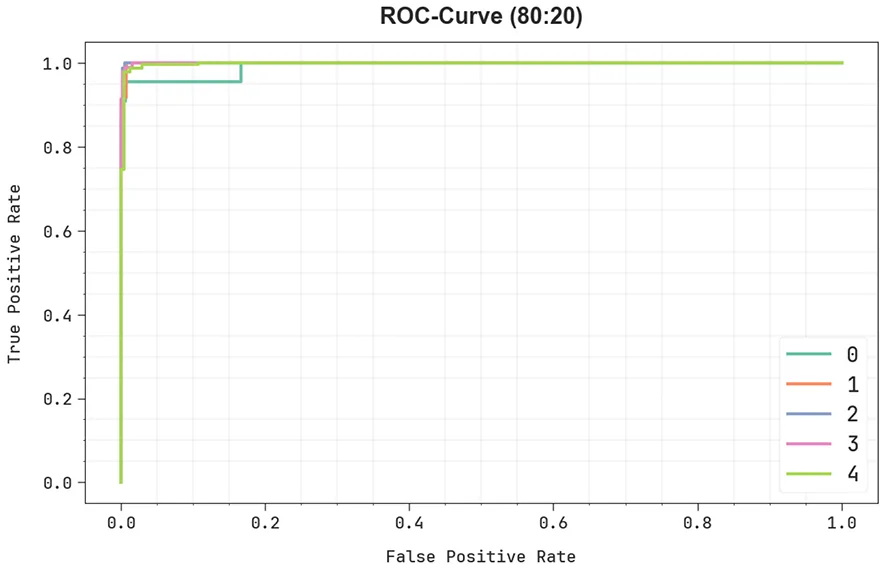
ROC curve of IAIMSA-SADMOA model under 80:20 in Dataset 1.

In Figure 8, the ROC curve of IAIMSA-SADMOA framework under epoch 80:20 is examined. The outcomes suggest that the proposed system achieves greater ROC values in all classes, establishing an important ability to distinguish the class labels. This consistent trend of superior values of ROC across many classes signifies the effective performance of the proposed framework in class prediction.

**Figure 9:**
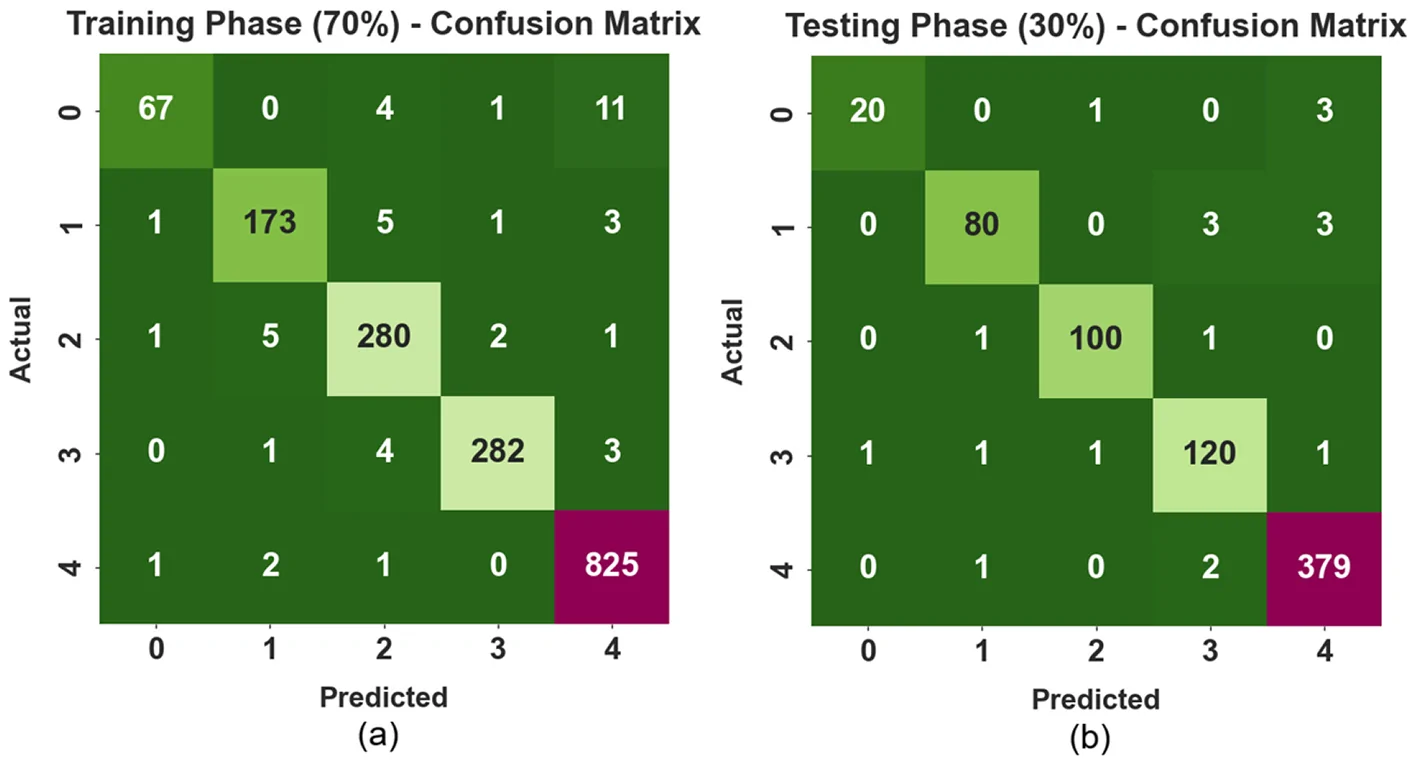
Confusion matrices **(a, b)** 70:30 of TRPHE/ TSPHE on the Dataset 1.

Figure 9 represents the confusion matrix for the training (70%) and testing (30%) sets on Dataset 1. In both phases, the maximum values are correctly classified, as shown by the high values confusion along the diagonal, while the few instances are misclassified into the various class labels. The training confusion metrics shows that the method significantly captures the class patterns, and the testing confusion matrix confirms that it keeps maximum classification accuracy and good generalization performance on unseen information.

Table 4 depict the students' performance prediction of IAIMSA-SADMOA technique under 70:30 of TRPHE/TSPHE. The solution indicated that the proposed methodology has properly classified every class label. Depending on 70% TRPHE, the proposed method reaches average *accu*_*y*_ of 98.88%, *prec*_*n*_ of 96.60%, *reca*_*l*_ of 93.78%, *F*_*Score*_ of 95.06%, and *AUC*_*score*_ of 96.47%. Likewise, under 30% TSPHE, the proposed system receives average *accu*_*y*_ of 98.94%, *prec*_*n*_ of 96.62%, *reca*_*l*_ of 94.08%, *F*_*Score*_ of 95.26%, and *AUC*_*score*_ of 96.63%.

**Table 4:** Students performance prediction of IAIMSA-SADMOA model under 70%:30% of TRPHE/TSPHE in Dataset 1.

Class labels	Accuracy	Precision	Recall	*F*-score	AUC score	MCC	Kappa score
TRPHE (70)							
0	98.86	95.71	80.72	87.58	90.27	87.34	95.85
1	98.92	95.58	94.54	95.05	97.00	94.45	
2	98.63	95.24	96.89	96.05	97.94	95.23	
3	99.28	98.60	97.24	97.92	98.48	97.49	
4	98.69	97.86	99.52	98.68	98.69	97.39	
Average	98.88	96.60	93.78	95.06	96.47	94.38	
TSPHE (30)							
0	99.30	95.24	83.33	88.89	91.59	88.74	95.92
1	98.75	96.39	93.02	94.67	96.27	93.98	
2	99.44	98.04	98.04	98.04	98.86	97.71	
3	98.61	95.24	96.77	96.00	97.88	95.16	
4	98.61	98.19	99.21	98.70	98.57	97.21	
Average	98.94	96.62	94.08	95.26	96.63	94.56	

**Figure 10:**
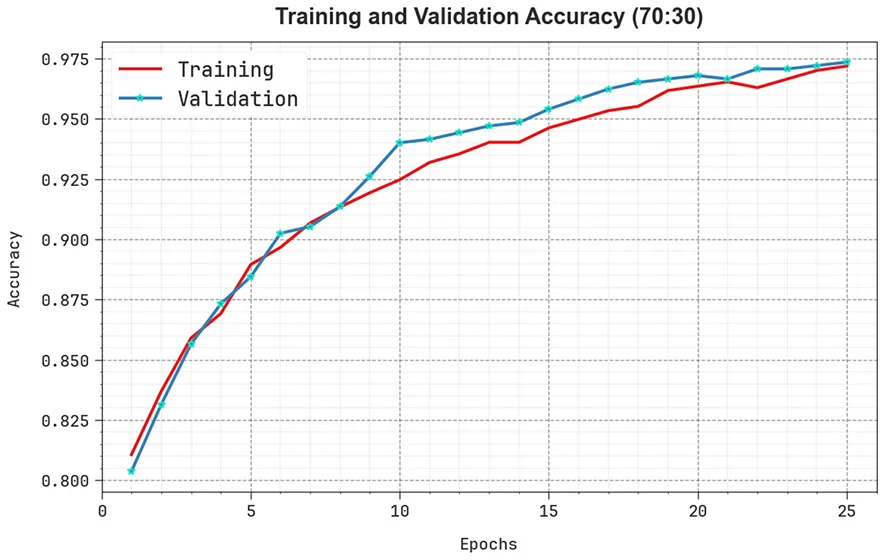
*Accu*_*y*_ curve of IAIMSA-SADMOA model under 70:30 with Dataset 1.

In Figure 10, the TRAN *accu*_*y*_ and VALD *accu*_*y*_ results of the proposed system under 70:30 is illustrated. The figure underlined that the TRAN and VALD *accu*_*y*_ values reveal maximal trends which stated the capability of the proposed methodology with enhanced outcomes through several iterations. Furthermore, the TRAN and VALD *accu*_*y*_ remains adjacent through the epochs, which represents lesser overfitting and shows superior results of the proposed system.

**Figure 11:**
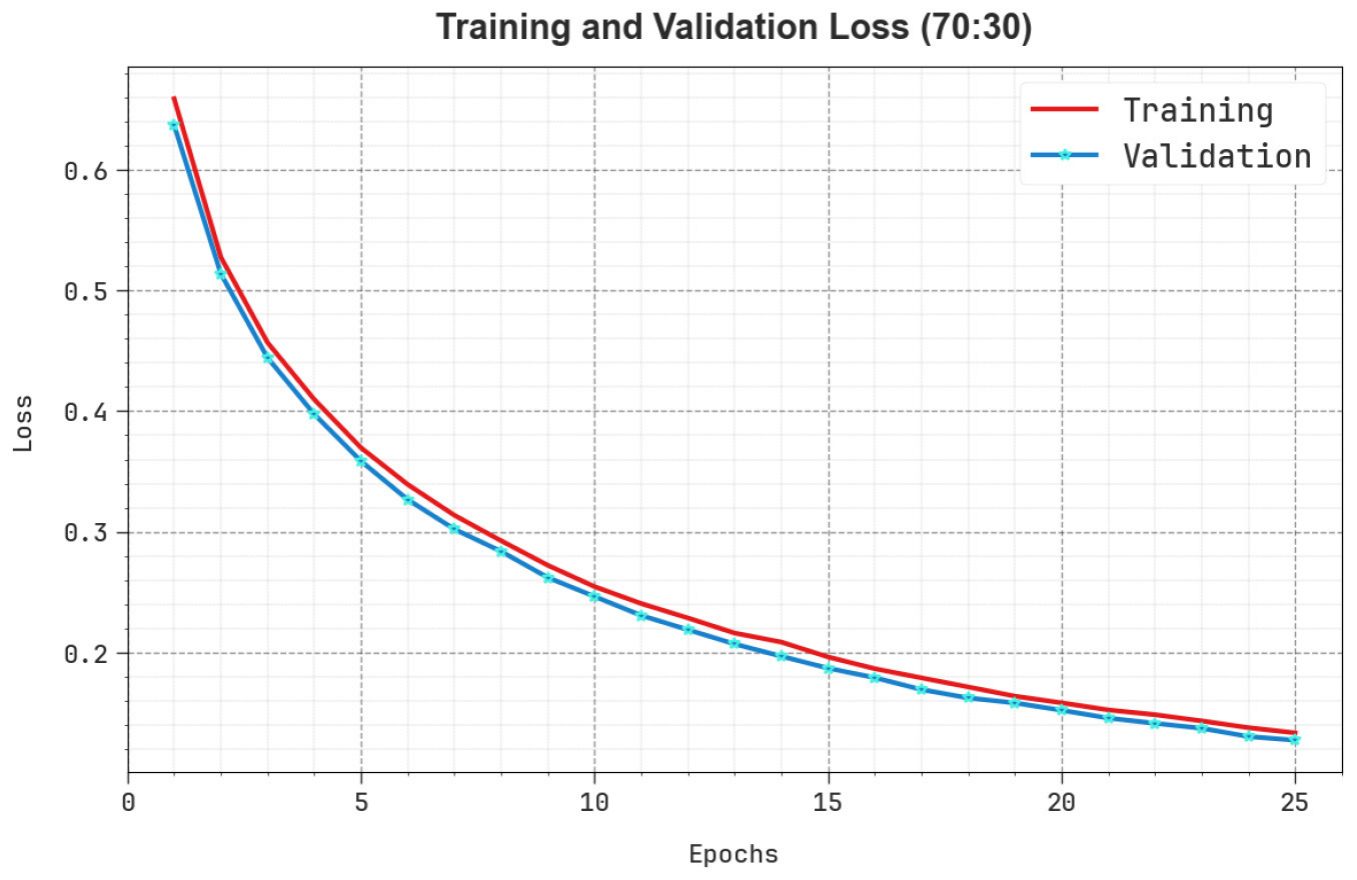
Loss curve of IAIMSA-SADMOA model under 70:30 in Dataset 1.

In Figure 11, the TRAN and VALD losses graph of IAIMSA-SADMOA approach under 70:30 is demonstrated. It is depicted that the TRAN and VALD values illustrate a minimal tendency, reporting the capability of the proposed methodology to equilibrium a trade-off. The constant decrease ensures the higher outcome of the proposed technique.

**Figure 12:**
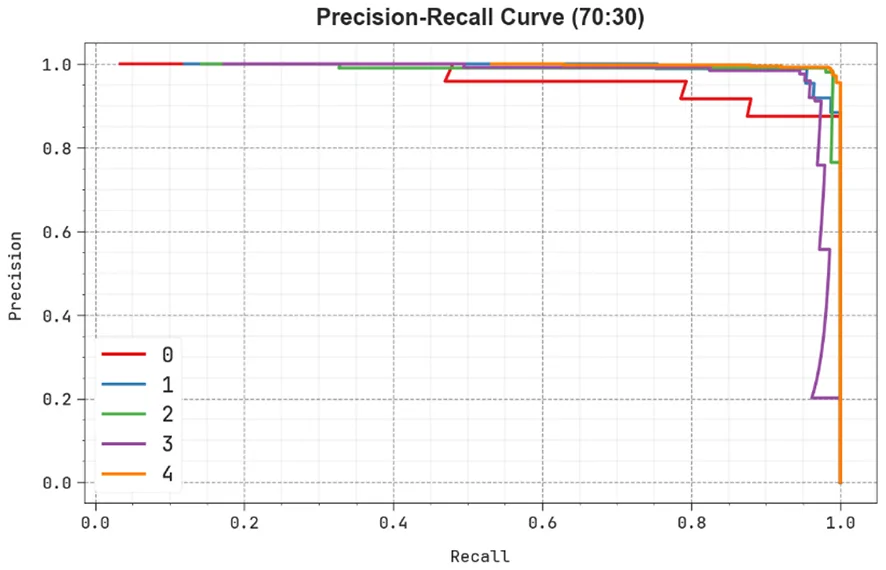
PR curve of IAIMSA-SADMOA model under 70:30 with Dataset 1.

In Figure 12, the PR curve inspection of the IAIMSA-SADMOA model under 70:30 provides interpretation of its outcomes by plotting precision against recall for every class label. The figure exposes that the proposed approach always reaches heightened values of PR through diverse classes, depicting its ability to preserve a substantial part of true positive predictions.

**Figure 13:**
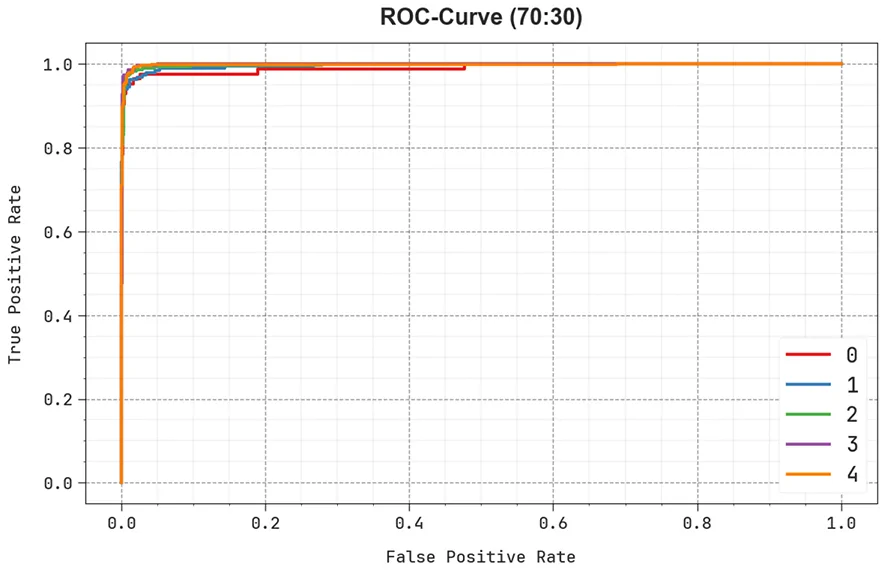
ROC curve of IAIMSA-SADMOA system under 70:30 on Dataset 1.

In Figure 13, the ROC curve of IAIMSA-SADMOA system under epoch 70:30 is examined. The solution suggests that the proposed technique achieves greater ROC solutions through every class, representing substantial proficiency in differentiating the labels. This consistent tendency of superior values of ROC through several class labels specifies the capable outcome of the proposed methodology on forecasting class labels.

### 4.2 Student performance analysis dataset (Dataset 2)

**Figure 14:**
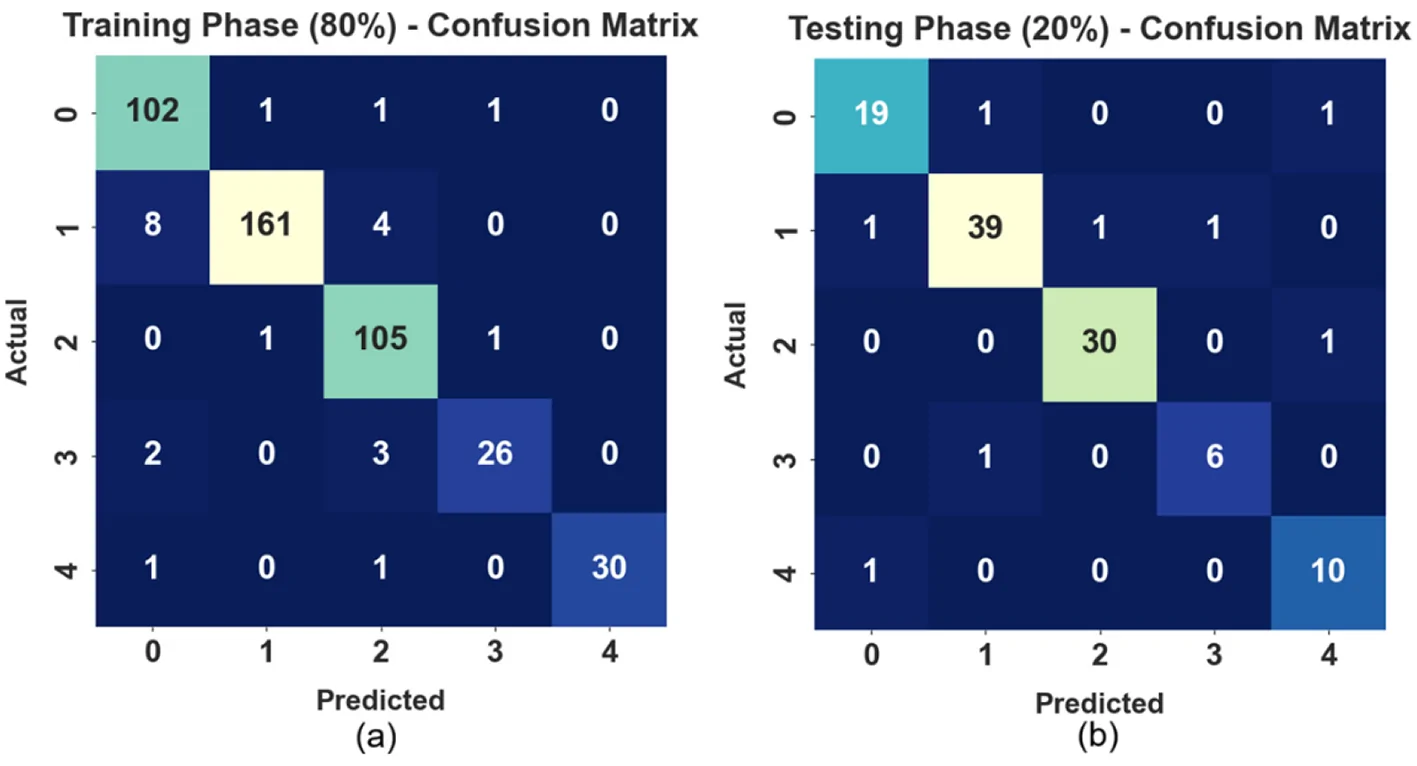
Confusion matrices **(a, b)** 80:20 of TRPHE/ TSPHE with Dataset 2.

Figures 14a, b depicts the confusion matrices of the proposed TRPHE/TSPHE technique on Dataset 2 utilizing an 80:20 training-testing split. The training confusion metrics provides that the algorithm correctly performed most instances over all five class labels, with only a slight misclassification among similar classes. Likewise, the testing confusion matrix illustrates better generalization ability, where the maximum of samples is accurately identified beside the diagonal. The minimum number of off-diagonal valued specifies that the proposed technique efficiently separates various classes with low classification errors.

Table 5 depicts the classification outcome of the IAIMSA-SADMOA method under 80:20. On 80% of TRPHE, the IAIMSA-SADMOA method gains average *accur*_*y*_, *preci*_*n*_, *recal*_*l*_, *F*_*score*_, MCC, and Kappa of 97.86%, 94.80%, 93.19%, 93.87%, 92.51%, and 92.67%, respectively. Also, on 20% of TSPHE, the IAIMSA-SADMOA method attains average *accur*_*y*_, *preci*_*n*_, *recal*_*l*_, *F*_*score*_, MCC, and Kappa of 97.14%, 90.28%, 91.35%, 90.78%, 88.92%, and 90.30%, correspondingly.

**Table 5:** Students performance analysis of IAIMSA-SADMOA model under 80%:20% of TRPHE/TSPHE in Dataset 2.

Classes	Accuracy	Precision	Recall	*F*-score	MCC	Kappa score
TRPHE (80%)							
0	96.88	90.27	97.14	93.58	91.62	92.67
1	96.88	98.77	93.06	95.83	93.44	
2	97.54	92.11	98.13	95.02	93.48	
3	98.44	92.86	83.87	88.14	87.43	
4	99.55	100.00	93.75	96.77	96.59	
Average	97.86	94.80	93.19	93.87	92.51	
TSPHE (20%)							
0	96.43	90.48	90.48	90.48	88.28	90.30
1	95.54	95.12	92.86	93.98	90.45	
2	98.21	96.77	96.77	96.77	95.54	
3	98.21	85.71	85.71	85.71	84.76	
4	97.32	83.33	90.91	86.96	85.57	
Average	97.14	90.28	91.35	90.78	88.92	

**Figure 15:**
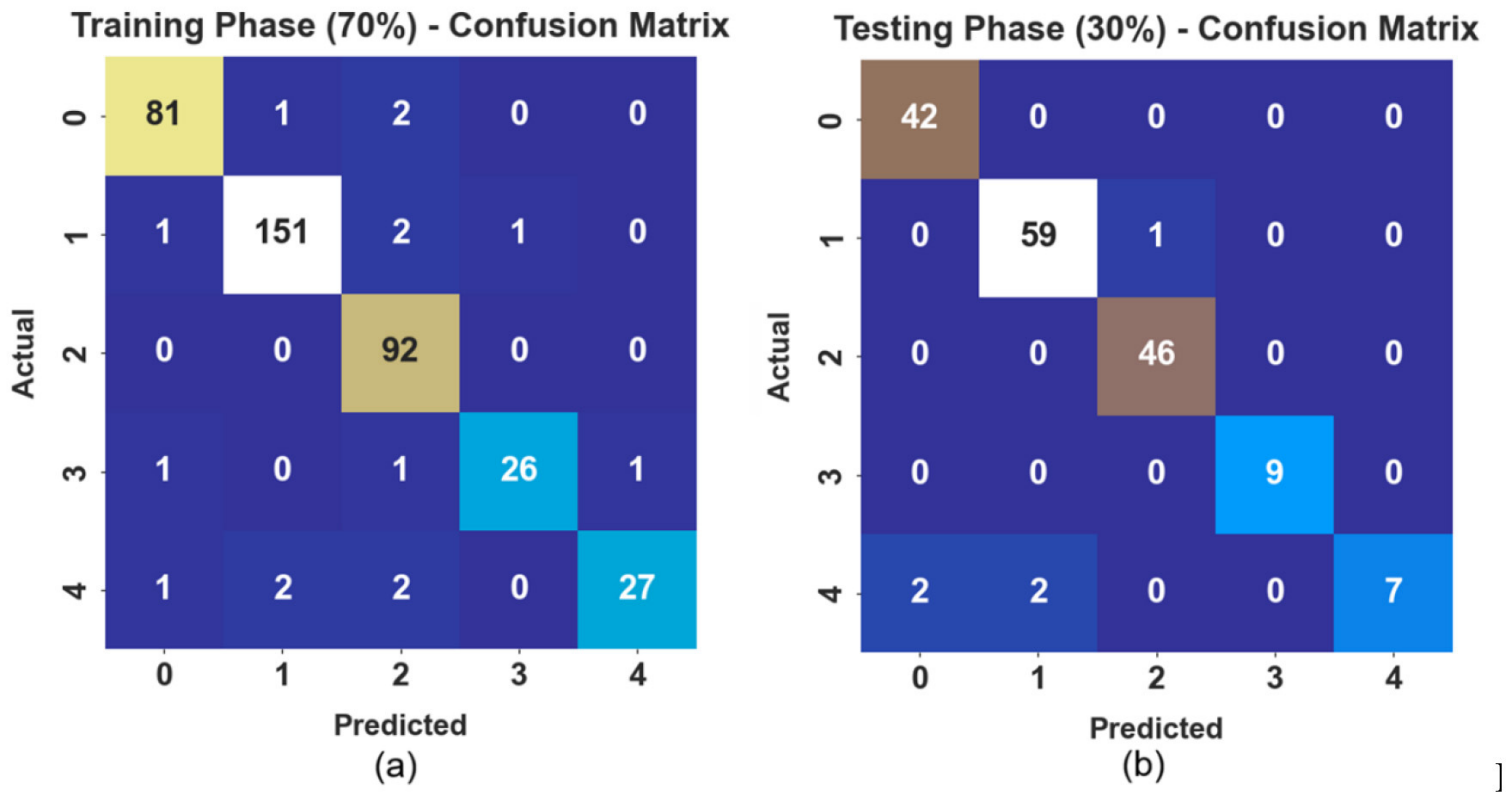
Confusion matrices **(a, b)** 70:30 of TRPHE/ TSPHE with Dataset 2.

Figure 15 illustrates the confusion matrices which show the classification performance of the proposed technique in the training (70%) and testing (30%). In two phases, maximum of the samples is correctly identified along the diagonal, demonstrating better discriminative capability across all five classes (0–4). In the training period, the method correctly classified 81, 151, 92, 26, and 27 instances of classes 0, 1, 2, 3, and 4, correspondingly, with only a few misclassifications between neighboring class labels. The minimum number of off-diagonal values illustrates that the approach reduces classification errors and maintains continuous performance on hidden testing data, confirming its generalization ability.

Table 6 illustrates the classifier analysis of the IAIMSA-SADMOA technique under 70:30. With 70% training, the IAIMSA-SADMOA approach achieves average *accur*_*y*_, *preci*_*n*_, *recal*_*l*_, *F*_*score*_, MCC, and Kappa of 98.47%, 96.03%, 93.58%, 94.67%, 93.75%, and 94.35%, respectively. Likewise, at 30% testing, the IAIMSA-SADMOA model attains average *accur*_*y*_, *preci*_*n*_, *recal*_*l*_, *F*_*score*_, MCC, and Kappa of 98.81%, 98.01%, 92.39%, 94.38%, 94.07%, and 95.88%, respectively.

**Table 6:** Students performance results of IAIMSA-SADMOA model under 70%:30% of TRPHE/TSPHE in Dataset 2.

Classes	Accuracy	Precision	Recall	*F*-score	MCC	Kappa score
Training phase (70%)							
0	98.47	96.43	96.43	96.43	95.45	94.75
1	98.21	98.05	97.42	97.73	96.26	
2	98.21	92.93	100.00	96.34	95.27	
3	98.98	96.30	89.66	92.86	92.38	
4	98.47	96.43	84.38	90.00	89.41	
Overall	98.47	96.03	93.58	94.67	93.75	
Testing phase (30%)							
0	98.81	95.45	100.00	97.67	96.92	95.88
1	98.21	96.72	98.33	97.52	96.13	
2	99.40	97.87	100.00	98.92	98.52	
3	100.00	100.00	100.00	100.00	100.00	
4	97.62	100.00	63.64	77.78	78.78	
Overall	98.81	98.01	92.39	94.38	94.07	

## 5 Discussion

This section discusses the classification performance of the proposed IAIMSA-SADMOA method on the dual datasets. The outcomes are recognized separately for each dataset to validate the efficiency, robustness, and generalization capability of the proposed method.

### 5.1 Results on the student performance analysis Dataset 1

To ensure unbiased and fair comparison, all baseline methods were assessed using the same benchmark dataset, same preprocessing procedures, and with the same training and testing partitions as the proposed framework. The performance settings of the comparative methods were selected from their corresponding original studies wherever applicable, and the respective references are provided. This protocol ensures the performance differences are accountable to the classification methods rather than the variations in preprocessing. Table 7 illustrates the comparative study of IAIMSA-SADMOA model with existing techniques under various metrics^2^ (Shou et al., 2024; Mai et al., 2024; Mohammad et al., 2023; Zhang et al., 2026; Yang et al., 2025). The table values emphasized that the current models, such as MTAPSP, TEnc, DLBRB-I, LR, ANN, Decision Tree, and GNB models have shown the worst performance. Whereas, the proposed model has obtained highest accuracy, precision, recall and *F*-score of 99.27 ± 0.23, 98.03 ± 0.73, 96.19 ± 4.17, and 97.06 ± 2.10 correspondingly.

**Table 7:** Comparative study of IAIMSA-SADMOA model with recent algorithms under Dataset 1.

Algorithm	Accuracy	Precision	Recall	*F*-score
MTAPSP	98.92 ± 0.31	97.57 ± 0.82	94.23 ± 1.95	90.61 ± 2.14
TEnc model	98.14 ± 0.42	91.31 ± 1.26	90.41 ± 1.58	96.72 ± 0.91
DLBRB-i	97.00 ± 0.56	96.19 ± 0.97	90.44 ± 1.87	90.83 ± 1.94
LR model	97.05 ± 0.48	94.47 ± 1.08	91.42 ± 1.52	93.25 ± 1.27
ANN method	98.30 ± 0.35	96.26 ± 0.88	94.19 ± 1.21	93.01 ± 1.44
Decision tree	94.44 ± 0.89	91.55 ± 1.74	95.59 ± 0.96	96.76 ± 0.81
GNB method	91.90 ± 1.08	90.43 ± 1.62	90.31 ± 1.75	95.43 ± 0.93
TabTransformer	72.48 ± 2.15	70.01 ± 2.48	71.96 ± 2.11	70.63 ± 2.32
TabNet	92.04 ± 0.97	80.89 ± 2.04	94.19 ± 1.18	87.03 ± 1.66
XGBoost	56.30 ± 3.14	57.60 ± 2.88	56.30 ± 3.05	54.20 ± 3.41
IAIMSA-SADMOA	99.27 ± 0.23	98.03 ± 0.73	96.19 ± 4.17	97.06 ± 2.10

In Table 8, the execution time (ET) of IAIMSA-SADMOA system with recent algorithms is proven. Based on ET, the proposed method provides a lower value of 6.66 s while the MTAPSP, TEnc, DLBRB-I, LR, ANN, Decision Tree, and GNB systems have greater ET of 24.70, 21.70, 17.34, 23.53, 14.62, 9.68, and 18.10 s, respectively.

**Table 8:** ET outcome of IAIMSA-SADMOA model with recent methods with Dataset 1.

Algorithm	Execution time (s)	CPU (%)	RAM (MB)	FLOPs (G)
MTAPSP	24.7	74.8	1,386	5.84
TEnc model	21.7	71.5	1,274	5.16
DLBRB-i	17.34	66.9	1,128	4.42
LR model	23.53	69.8	1,216	4.91
ANN method	14.62	61.4	948	3.56
Decision tree	9.68	49.2	612	1.42
GNB method	18.1	58.6	784	2.18
IAIMSA-SADMOA	6.66	41.8	486	0.94

Table 9 represents the ablation study of the proposed IAIMSA-SADMOA framework by evaluating the contribution of each major component. The BiLSTM only model achieved an accuracy of 96.74%, precision of 95.58%, recall of 92.64%, *F*1-score of 94.09%, AUC score of 95.68%, MCC of 92.48%, and Kappa score of 93.16%. However, the BiLSTM + MSA model improved the performance by obtaining an accuracy of 97.65%, precision of 96.44%, recall of 93.98%, *F*1-score of 95.19%, AUC score of 96.52%, MCC of 93.84%, and Kappa score of 94.67%, demonstrating the effectiveness of the Multi-Head Self-Attention mechanism. Finally, the full proposed framework attained the highest performance with an accuracy of 99.27%, precision of 98.03%, recall of 96.19%, *F*1-score of 97.06%, AUC score of 97.82%, MCC of 96.57%, and Kappa score of 97.26%, demonstrating that the integration of all components provides the best overall classification performance.

**Table 9:** Ablation study of IAIMSA-SADMOA model.

Method	Accuracy	Precision	Recall	*F*-score	AUC score	MCC	Kappa score
BiLSTM only	96.74	95.58	92.64	94.09	95.68	92.48	93.16
BiLSTM + MSA	97.65	96.44	93.98	95.19	96.52	93.84	94.67
FOA + BiLSTM	98.12	97.08	94.82	95.94	97.05	94.85	95.61
BiLSTM + SSA	98.58	97.61	95.46	96.52	97.46	95.78	96.43
IAIMSA-SADMOA	99.27	98.03	96.19	97.06	97.82	96.57	97.26

Table 10 represents the performance of the proposed model using 10-fold cross-validation. In Fold 1, the proposed model achieved an accuracy of 99.05%, precision of 97.84%, recall of 95.71%, *F*1-score of 96.76%, AUC score of 97.48%, MCC of 96.18%, and Kappa score of 96.83%. Moreover, Fold 8 recorded an accuracy of 99.24%, precision of 98.02%, recall of 96.15%, *F*1-score of 97.08%, AUC score of 97.80%, MCC of 96.55%, and Kappa score of 97.22%, demonstrating highly consistent predictive capability. Finally, Fold 10 attained an accuracy of 99.23%, precision of 98.02%, recall of 96.12%, *F*1-score of 97.06%, AUC score of 97.79%, MCC of 96.54%, and Kappa score of 97.20%, illustrating the consistent and reliable performance of the proposed model across different data partitions. Additionally, the mean values of 99.17% accuracy, 97.96% precision, 95.95% recall, 96.95% *F*1-score, 97.67% AUC Score, 96.40% MCC, and 97.06 Kappa score specify the continuous performance of the proposed IAIMSA-SADMOA method over all folds. The corresponding standard deviations (±SD) of 0.06, 0.06, 0.14, 0.10, 0.10, 0.12, and 0.13, correspondingly, illustrate minimum performance difference, confirming the model's reliability and robustness.

**Table 10:** *K*-fold cross-validation results of the IAIMSA-SADMOA system.

Fold	Accuracy	Precision	Recall	*F*1-score	AUC score	MCC	Kappa score
Fold 1	99.05	97.84	95.71	96.76	97.48	96.18	96.83
Fold 2	99.12	97.91	95.82	96.85	97.56	96.29	96.94
Fold 3	99.18	97.98	95.96	96.96	97.68	96.42	97.08
Fold 4	99.20	98.00	96.02	97.00	97.71	96.46	97.12
Fold 5	99.16	97.95	95.88	96.90	97.63	96.34	97.00
Fold 6	99.22	98.01	96.10	97.05	97.76	96.52	97.18
Fold 7	99.14	97.93	95.85	96.88	97.59	96.31	96.98
Fold 8	99.24	98.02	96.15	97.08	97.80	96.55	97.22
Fold 9	99.17	97.96	95.93	96.93	97.66	96.39	97.05
Fold 10	99.23	98.02	96.12	97.06	97.79	96.54	97.20
Mean	99.17	97.96	95.95	96.95	97.67	96.40	97.06
SD	±0.06	±0.06	±0.14	±0.10	±0.10	±0.12	±0.13

### 5.2 Results on the student performance analysis Dataset 2

Table 11 illustrates the comparative study of IAIMSA-SADMOA model with existing techniques under various metrics (Qiang et al., 2026). The values in the table emphasizes that the current models, such as SVM, LR, GaussianNB, VAE-SVM, AE-SVM, and GNN-S models have shown the worst performance. Whereas, the proposed model has obtained highest accuracy, precision, recall and F-score of 98.81, 98.01, 92.39, and 94.38 correspondingly.

**Table 11:** Comparative study of IAIMSA-SADMOA model with recent algorithms under Dataset 2.

Methods	Accuracy	Precision	Recall	F1-score
SVM	87.92	85.51	91.17	90.08
LR	90.18	90.34	87.21	88.75
GaussianNB	89.26	87.21	91.42	89.27
VAE-SVM	88.95	85.27	88.44	86.83
AE-SVM	89.91	93.84	89.30	91.51
GNN-S	90.63	88.93	91.69	90.29
IAIMSA-SADMOA	98.81	98.01	92.39	94.38

Table 12 demonstrates the computation efficiency of IAIMSA-SADMOA method with other algorithms. Based on execution time, the IAIMSA-SADMOA approach gives minimum value of 3.18 s, Flops of 0.71G and CPU of 34.91%, while the VAE-SVM, AE-SVM, and GNN-S method reaches maximum time of 3.18, 2.67, and 4.26 s, correspondingly.

**Table 12:** Computational efficiency of IAIMSA-SADMOA technique.

Methods	Execution time (s)	CPU (%)	RAM (MB)	FLOPs (G)
VAE-SVM	3.18	57.41	412.89	2.37
AE-SVM	2.67	53.06	374.28	2.08
GNN-S	4.26	64.35	598.73	4.91
IAIMSA-SADMOA	1.18	34.91	176.82	0.71

An ablation study explains how dissimilar role of a method impacts its performance by testing the method with certain module eliminated or changed. This procedure provided which module are significant and which have less impact. By comparing results, investigators obtain understanding into the model's behavior. These results support better plan and optimization decisions. Table 13 depicts the ablation study of IAIMSA-SADMOA model. The results specify that BiLSTM only, BiLSTM + MSA, and FOA + BiLSTM, BiLSTM + SSA techniques have attained minimum performance under different metrics. In the meantime, the IAIMSA-SADMOA method has obtained maximum results with *accur*_*y*_, *preci*_*n*_, *recal*_*l*_, *F*_*score*_, MCC, and Kappa of 98.81%, 98.01%, 92.39%, 94.38%, 94.07%, and 95.88%, correspondingly.

**Table 13:** Ablation study analysis of the proposed IAIMSA-SADMOA algorithm.

Method	Accuracy	Precision	Recall	*F*-score	MCC	Kappa score
BiLSTM only	93.46	92.08	84.73	88.25	87.62	90.14
BiLSTM + MSA	95.24	94.36	88.41	91.29	90.87	92.83
FOA + BiLSTM	96.31	95.18	89.86	92.44	92.31	93.74
BiLSTM + SSA	97.42	96.62	91.14	93.8	93.36	94.81
IAIMSA-SADMOA	98.81	98.01	92.39	94.38	94.07	95.88

Table 14 represents the *k*-fold cross-validation results of the proposed IAIMSA-SADMOA technique. The algorithm obtained accuracy values ranged from 95.84% to 97.32%, with the high-performance accuracy of 97.32% in CV 8. The better precision of 97.13%, recall of 91.44%, and *F*1-score of 93.82% were reached in *k*-7. While slight variations over the folds, the continuously high validation metrics demonstrates the robustness, reliability, and strong generalization capability of the proposed technique. Additionally, the mean and standard values of 96.68 ± 0.46, 96.25 ± 0.50, 90.64 ± 0.58, and 93.29 ± 0.48 values specify the continuous performance of the proposed IAIMSA-SADMOA method through all folds.

**Table 14:** *K*-fold cross validation results of IAIMSA-SADMOA method.

*K*-fold	Accuracy	Precision	Recall	F1-Score
*k*-1	95.84	95.32	89.66	92.40
*k*-2	96.91	96.44	90.82	93.55
*k*-3	96.37	95.88	90.18	92.94
*k*-4	97.08	96.73	91.08	93.56
*k*-5	96.58	96.05	90.47	93.18
*k*-6	96.82	96.39	90.95	93.59
*k*-7	97.32	97.13	91.44	93.82
*k*-8	96.14	95.67	89.98	92.74
*k*-9	96.74	96.28	90.61	93.36
*k*-10	97.03	96.59	91.16	93.80
Mean ± SD	96.68 ± 0.46	96.25 ± 0.50	90.64 ± 0.58	93.29 ± 0.48

The high performance of the proposed model is attributed to the integrated strengths of SSA-based hyperparameter optimization, BiLSTM-multi-head self-attention, and FOA-based feature selection. It can support in the early identification of students demanding academic assistance, facilitates timely educational interventions.

## 6 Conclusion

This study has presented the IAIMSA-SADMOA model which aims to advance an effective method for students' academic performance using AI tools to improve learning outcomes, engagement, and personalized education. Primarily, the min–max normalization is employed in the data pre-processing phase for converting an input data into a beneficial format. Then, the FOA is deployed for the feature selection process. Moreover, the proposed model designs BiLSTM-MSA mechanism for the student's academic performance classification process. Lastly, the parameter tuning process is achieved through SSA to enhance the performance of classification. The experimental evaluation of the proposed model occurs using benchmark dataset. The empirical results indicated the greater performance of the IAIMSA-SADMOA system in comparison with recent methodologies.

Although the proposed model indicates high performance, still remains a constraint of Kaggle dataset which was collected before the extensive adoption of GenAI tools in the educational settings. Hence, it may not fully reflect the impact of AI-based learning on students' academic performance. In future, research should assess the proposed framework by more recently adopted educational datasets which captures the influence of GenAI in assessing practical applicability and generalizability. Future work can focus on explainability techniques like SHAP analysis, attention visualization, and feature importance evaluation to provide deeper perceptions into the factors which influence the students' academic performances. Additionally, future study will be based on class-imbalance handling approaches, such as class weighting, focal loss, and training-fold-based resampling, to enhance minority-class prediction outcome. It will be explored in future works to improve transparency and practical adoption of the proposed architecture in educational decision making.

## Data Availability

The data that support the findings of this study are openly available in Kaggle dataset https://www.kaggle.com/datasets/rabieelkharoua/students-performance-dataset and https://www.kaggle.com/datasets/waleedejaz/predict-students-dropout-and-academic-success/data, reference number [23, 24].

## Footnotes

1 Available online at: https://www.kaggle.com/datasets/rabieelkharoua/students-performance-dataset.

2 Available online at: https://data.mendeley.com/datasets/vzfyk22fhn/4.
